# Precision and practical usefulness of intraoral scanners in implant dentistry: A systematic literature review

**DOI:** 10.4317/jced.57025

**Published:** 2020-08-01

**Authors:** Ignacio García-Gil, Jorge Cortés-Bretón-Brinkmann, Jaime Jiménez-García, Jesus Peláez-Rico, María-Jesús Suárez-García

**Affiliations:** 1 DDS, MS. Master Program Advanced Oral Implantology Europea University of Madrid. Master Program Buccofacial Prostheses and Occlusion, Faculty of Dentistry, Complutense University of Madrid; 2 DDS, PhD, MS. Researcher/Assistant Professor of Oral Surgery and Buccofacial Prostheses, Faculty of Dentistry, Complutense University of Madrid; 3 DDS, PhD, MS. Full profesor and Chairman. Implantology Department, European University of Madrid. Surgical Director of CIRO, Madrid; 4 DDS, PhD, MS. Assistant Professor. Department Conservative Dentistry and Buccofacial Prostheses, Faculty of Dentistry, Complutense University of Madrid; 5 MD, DDS, PhD, MS. Full Professor. Department Conservative Dentistry and Buccofacial Prostheses, Faculty of Dentistry, Complutense University of Madrid

## Abstract

**Background:**

This systematic review aimed to evaluate the efficiency and accuracy of digital impression techniques for implant-supported restorations, and to assess their economic feasibility.

**Material and Methods:**

Two independent electronic database searches were conducted in the Pubmed/MedLine, Cochrane Library, and Lilacs databases complimented by a manual search, selecting relevant clinical and *in vitro* studies published between 1st January 2009 and 28st February 2019. All type of studies (*in vivo* and *in vitro*) were included in this systematic review.

**Results:**

Twenty-seven studies (8 *in vivo* and 19 *in vitro* studies) fulfilled the inclusion criteria. No meta-analysis was performed due to a large heterogeneity of the study protocols. The passive fit of superstructures on dental implants presented similar results between digital and conventional impression techniques. The studies considered that several factors influence the accuracy of implant impression taking: distance and angulation between implants, depth of placement, type of scanner, scanning strategy, characteristics of scanbody, and operator experience. Regarding the economic viability of intraoral scanning systems, only one study reported any benefit in comparison with conventional techniques.

**Conclusions:**

Digital impressions of dental implants can be considered a viable alternative in cases of one or two contiguous dental implants. However, more studies are needed to evaluate the accuracy of digital techniques in full-arch implant-supported restorations.

** Key words:**Intraoral scanner, dental implant, prosthesis, misfit, systematic review.

## Introduction

It is many years since the long-term success of dental implants was confirmed by Branemark *et al.* and Albrektsson *et al.* (1,2) Since then, numerous studies have described new surgical and prosthodontic techniques that aim to improve the clinical outcomes of implant-based treatments (3,4). In cases of implant-supported restorations, treatment success depends on the superstructure’s passive fit, as failure to achieve adequate passive fit can produce biological and mechanical complications (5). Fit depends on the accuracy of implant impression taking, which may be realised using long-established conventional techniques or more recently introduced digital techniques. The fabrication of an implant-supported prosthesis in a conventional workflow must start with the aid of an implant transfer post. Conventional impression taking can be classified as direct (pick-up) or indirect (transfer).

With the introduction of digital technologies in dentistry, intraoral scanners can now be used for digital impression taking. According to the manufacturers, the use of intraoral scanners are a key element in the digital workflow, providing greater comfort for the patient, decreased turnaround time, and even a better cost-benefit ratio when compared to conventional techniques (6). But to date, no systematic literature review has been conducted to confirm the advantages of digital impression taking. In this context, this systematic literature review aimed to: (a) to determine if it is possible to achieve an adequate level of accuracy and efficiency using intraoral digital impression systems and to compare them with various conventional techniques for implant-supported restorations and (b) to assess the economic feasibility of digital techniques.

## Material and Methods

This systematic review was conducted following PRISMA guidelines (7) and was registered in the Prospero database (trial no. CRD42015029504). The systematic review focused question was based on the PICO format (Population, Intervention, Comparison, Outcome) as follows.

Population: healthy adult human patients.

Intervention: conventional impression techniques.

Comparison: digital impression taking with intra-oral scanners.

Outcome: accuracy of impression and efficiency for fixed implant-supported restorations.

-Study Selection Criteria

In order to identify relevant articles, the following inclusion criteria were applied: Clinical studies without language restriction that evaluated the accuracy of digital impressions taken with intraoral scanners or compared digital impression taking with conventional impression taking in treatment protocols leading to fixed implant-supported restorations. As the initial search generated only a few articles, and so insufficient scientific evidence, the search was extended to include *in vitro* studies. Finally, due to the heterogeneity of different articles it was not possible implement a meta-analysis.

-Search Strategy 

An electronic search was conducted in the following databases: PubMed, Cochrane Library, Lilacs. Key search terms were applied, combined using MesH terms, to locate relevant articles published between 1st January 2009 and 28st February 2019. A additional manual search was conducted in the following journals: Clinical Implant Dentistry and Related Research, International Journal of Oral & Maxillofacial Implants, Journal of Oral Implantology, Clinical Oral Implants Research, Journal of Dental Research, Clinical Oral Implants Research, European Journal of Oral Implantology, Implant Dentistry, International Journal of Oral and Maxillofacial Surgery, Journal of Oral Implantology, Journal of Dentistry, Clinical Oral Investigations, and Journal of Oral Rehabilitation. All the corresponding authors of the studies identified were contacted in order to ascertain if additional articles or unpublished data were available.

-Data Collection and Quality Assessment 

The search was carried out by two independent reviewers. Any disagreement between the reviewers (IGG and JC-BB) regarding data collection or quality assessment was resolved by consensus. Inter-reviewer reliability was assessed obtained a Kappa coefficient of 0.88 (CI 95%), values above 0.8 being considered a good level of agreement (8). To assess the quality of *in vivo* articles, the Critical Appraisal Skills Program (CASP) proposed by the Public Health Resource Unit (2006) was used, and only studies with an overall score of at least 50% were included in the review. Due to the small number of *in vivo* studies available, a duplicate search was performed to obtain *in vitro* studies. Although *in vitro* research cannot reproduce the dynamic environment of the stomatognathic system or human variability, pre-clinical experiments can provide important information about the properties and characteristics of a new material or technique. It is therefore necessary to conduct *in vitro* research of the highest possible standard. Efforts have been made in recent years to improve the quality of reporting in scientific literature (9,10). Although the CASP consort checklist was not originally designed for analyzing *in vitro* trials, in 2012 a modified consort checklist was published of items selected to assess reporting *in vitro* studies of dental materials.18 The authors of the present review adapted this checklist for the purpose of comparing the accuracy of different dental implant impression-taking techniques. Only studies with an overall score of at least 50% were included in the review.

## Results

-Included Studies

**Figure 1:**
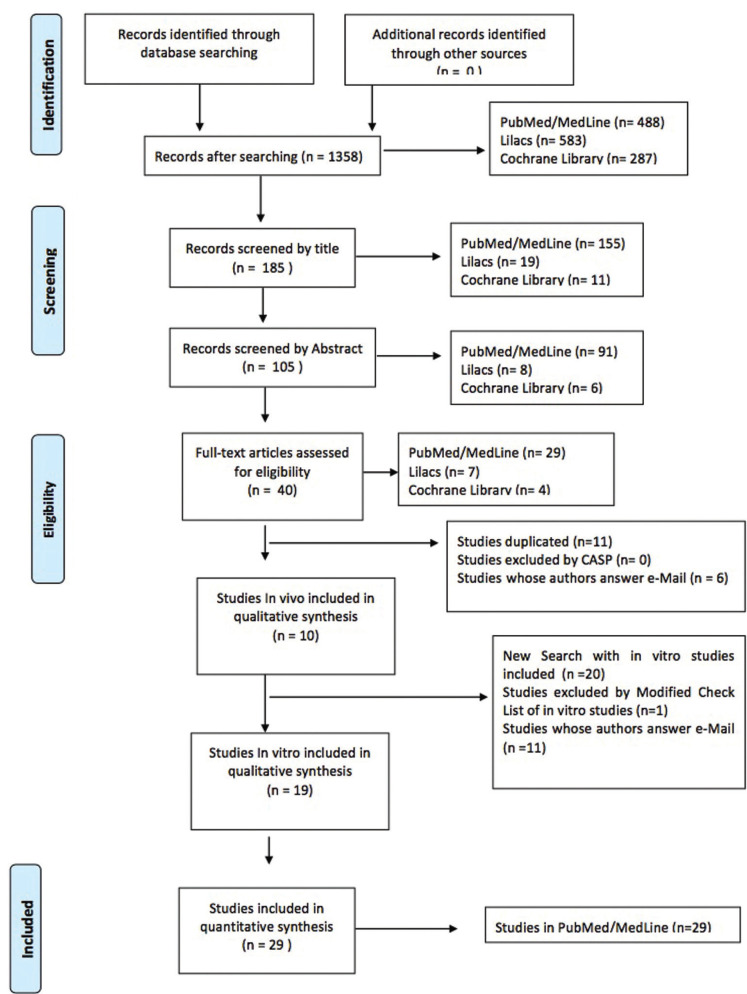
Numbers of articles in databases.

An electronic search of the PubMed/MedLine, Cochrane Library and Lilacs databases located 1358 articles, which were reduced to 40 following title, abstract and full text analysis (PubMed/MedLine n=29; Lilacs n=7; Cochrane Library n=4). The articles from the different databases were compared to identify any duplicates, and a further 11 articles were eliminated on the basis of duplication (n=11) (Fig. 1). The ten remaining *in vivo* articles were categorized as follows: systematic reviews (n=5), randomized clinical trials (RCT) (n=1), prospective cohort studies (n=1), case-control studies (n=2), and case reports (n=1).

**Figure 2:**
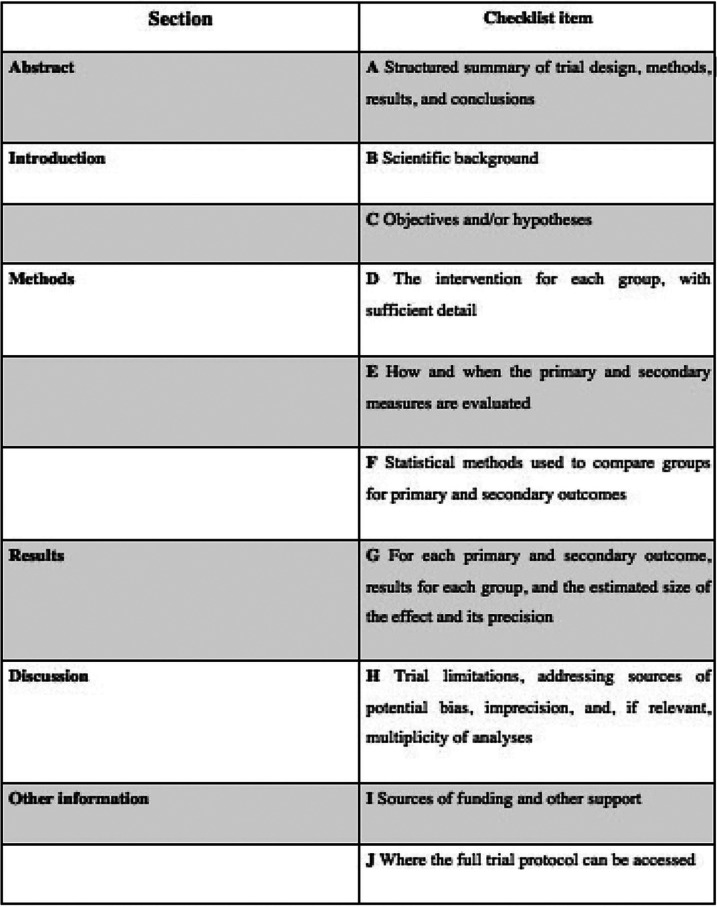
Modified consort Checklist *in vitro* studies.

The corresponding authors of the selected studies were contacted via email of whom four returned additional data. However, no additional data was included for analysis as all proved to be either replicate information or failed to meet the inclusion criteria. Due to the small number of *in vivo* studies available, the search was extended to include *in vitro* studies, using the same method, selecting 20 additional *in vitro* studies. These authors were also contacted via email, generating further data in three cases (n=3), but these were not included in the review for the same reasons as before. A modified CONSORT checklist of items for reporting *in vitro* studies was used to evaluate the risk of bias in the *in vitro* studies included (Fig. 2). When applying this modified CONSORT checklist to *in vitro* articles, points 5-9 could not be applied as they were designed to evaluate sample standardization. In the *in vitro* studies, the master model was the same in each study group, and so always standard. Of the articles evaluated, only one19 did not exceed the minimum score for inclusion in the review (5/10), obtaining a score of 0/10 and so was eliminated (Table 1). Finally, the review included eight *in vivo* and 19 *in vitro* studies. The reasons for exclusion of various articles are specified in (Table 2).

**Table 1:**
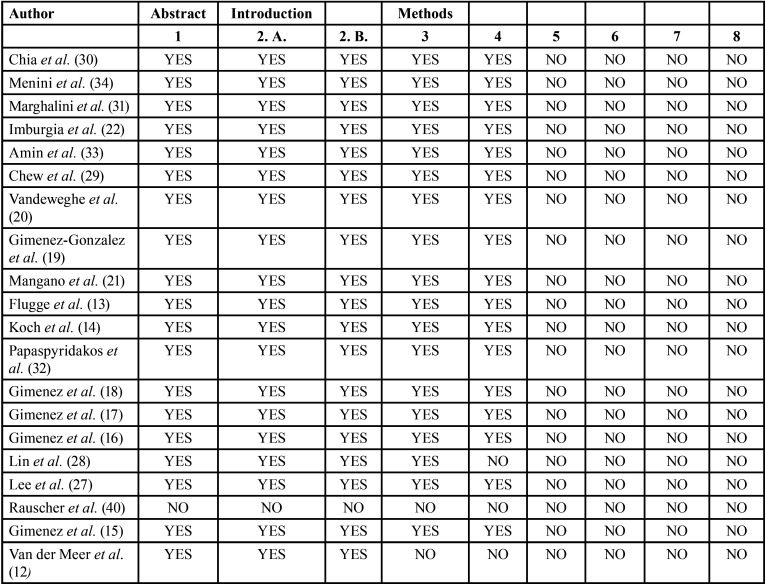
Modified checklist used to assess quality and risk of bias of *in vitro* studies.

**Table 2:**
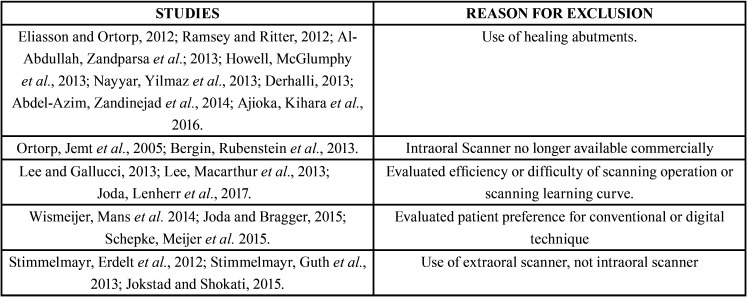
Studies excluded and reasons for exclusion.

-Outcomes

Implant impressions can be obtained using open or closed tray, with or without splinting, using different impression materials (CI) or scanbody + an intraoral scanner system (DI). In order to carry out a complete analysis of the included articles, the outcomes were divided according to the technique(s) investigated: DI (17 studies), or CI vs. DI (12 studies) ( Table 3, Table 4).

**Table 3:**
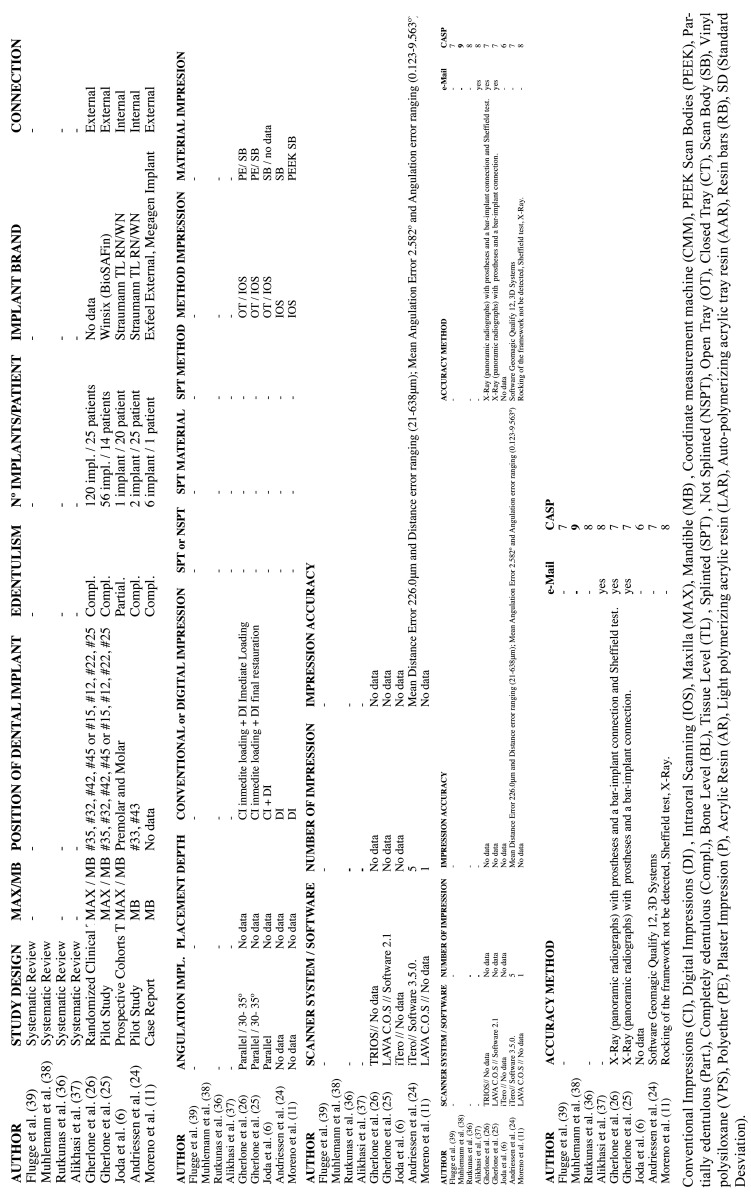
*In vivo* studies.

**Table 4:**
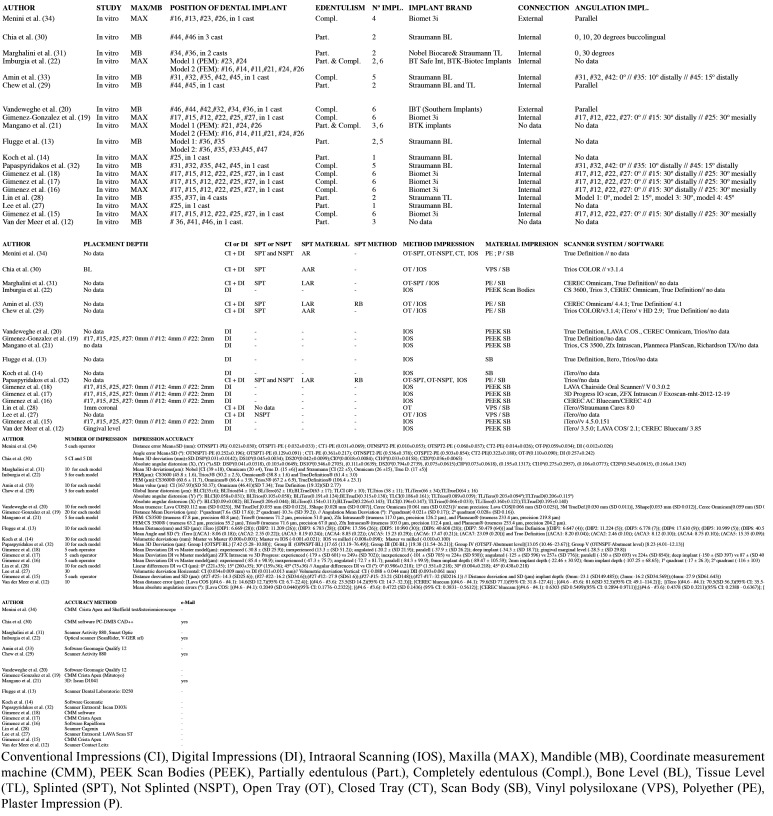
*In vitro* studies.

1. DI

Seventeen studies used DI to take impression of dental implants: five systematic reviews, one case report, and eleven *in vitro* studies.

In Vivo

This case report describes DI in a patient with a fully edentulous jaw rehabilitated with six dental implants; three clinical tests were carried out to evaluate the accuracy of the superstructure: saliva intrusion, the Sheffield test, and the screw resistance test, although the authors did not specify the fit values obtained (11).

In Vitro

Eleven *in vitro* studies were located that investigated the accuracy of IOS, divided into three subgroups: partially edentulous (PE), completely edentulous (CE), and partially and completely edentulous models (CE-PE).

*In Vitro* - PE

Three *in vitro* studies used DI-PE models (12-14).

In 2012, Van der Meer *et al.* (12) carried out a study using a PE model with the aim of evaluating the accuracy of three different IOS. The authors concluded that the Lava COS was more accurate than the other IOS. Flugge *et al.* (13) employed two models bearing dental implants to compare the precision of three IOS with a laboratory scanner, obtaining a decrease in precision of the IOS when the distance between scan bodies increased, whereas with the dental lab scanner this was not dependent. Koch *et al.* (14). compared volumetric deviations between single tessellation language (STL) datasets of a master model, and milled model, and IOS from a previous single implant model. The authors concluded that direct digitization using the IOS presented less systematic error than physical model fabrication by milling from IOS.

*In Vitro* - CE

Six *in vitro* studies used digital techniques to scan CE models (15-20).

In the studies carried out by Giménez *et al.*, (15-19) precision was assessed in an edentulous maxillary model with different implant angulations.The same authors (15) concluded that the accuracy of impressions with iTero® IOS (Cadent) decreased with the increased length of the scanned section but the angulation of dental implants did not affect scanning accuracy. In 2015, Giménez *et al.* (18) performed a study to assess the accuracy of two different IOS: ZFX Intrascan® (Zimmer Biomet, Dachau Germany) and 3D Progress® (MHT, Verona, Italy), concluding that neither IOS was suiTable for taking impressions of dental implants in the full arch. In the same way, Giménez *et al.* (17) concluded that angulated and deep implant placement did not seem to decrease the system’s accuracy with Lava COS® intraoral scanning system (3M ESPE), although accuracy was higher among experienced operators. Also in 2015, the same authors published another *in vitro* study of the CEREC AC Bluecam (Sirona) intraoral scanner. They concluded that neither angulation nor implant depth significantly affected scanner accuracy but operator experience did, with a tendency for less experienced operators to commit lower levels of error (16). In 2017, Giménez-González *et al.* (19) concluded that 3M True Definition IOS (3M ESPE) allows impression taking within the clinically accepTable range *in vitro*, and they identified certain factors that influence accuracy: the amount of visible scanbody, distance and angulation between scan bodies; and operator experience. Vandeweghe *et al.* (20) carried out a study to evaluate the accuracy (trueness and precision) of four IOS in a mandibular model. The authors concluded that the 3M True Definition (3M ESPE) and Trios (3Shape) scanners presented accepTable levels of trueness and precision for dental implant impression taking, but that LAVA COS (3M ESPE) failed to obtain the minimum level of accuracy.

*In Vitro* –PE-CE

Two *in vitro* studies used digital techniques in (PE) and completely (FE) models (21,22).

Mangano *et al.* (21) used two models (PEM and FEM) and four IOS. No differences were found in trueness and precision between the IOS; however, differences were found between the PEM and FEM with different IOS. In 2017, Imburgia *et al.* (22) also carried out a study with PEM and FEM, concluding that scanning with IOS was more accurate on the PEM than the FEM, findings that could have important clinical implications.

2. CI vs DI

The twelve articles that compared (CI) with (DI) included four *in vivo* and eight *in vitro* studies.

In Vivo

Comparisons between CI and DI were analyzed in four *in vivo* studies: a randomized crossover trial (23), two pilot studies (24,25), and one randomized clinical trial (26). Andriessen *et al.* (24) assessed the accuracy of IOS (iTero) in edentulous mandibles rehabilitated with overdentures compared with an extraoral laboratory scanner. They concluded that inter-implant distance and implant angulation were critical factors influencing the accuracy of intraoral scanning. Gherlone *et al.* (25) carried out two cases series studies with a similar design: CE rehabilitated with the “All on Four” protocol. In 2015, CI and DI (LAVA C.O.S scanner, 3M ESPE) were performed, assessing the accuracy of metallic structures through the use of an X-Ray (intraoral digital radiographs). In 2016, the patients were allocated either to the control group (CI) or test group (DI, using the Trios (3Shape). The authors concluded that it is possible to manufacture cobalt-chromium full-arch rehabilitations using computer-aided design/computer-assisted manufacturing (CAD/CAM) from DI with satisfactory accuracy (26). Joda *et al.* (23) concluded that in addition to the multiple benefits offered by digital technology, DI allows a more efficient workflow in terms of cost when compared with CI.

In Vitro

The present review included eight *in vitro* studies divided into two subgroups: PE (27-31) and CE (32-34).

*In Vitro* - PE

Lee *et al*. (27) compared the models obtained with CI and DI, using a PE customized maxillary model. The authors reported that there were no statistically significant differences between DI and CI, although statistically significant differences were found with the reference model. Lin *et al.* (28) used four different models with dental implants placed with varying angulation, fabricating definitive casts, observing a decreasing linear trend in deviations for both distance and angle measurements, suggesting that DI was more accurate when the implants diverged more. Marghalini *et al.* (31) found, in their study, which compared CI and DI, that impression techniques could affect accuracy, although within clinically accepTable levels.

Chew *et al.* (29) also evaluated this parameter in two sectional mandibular arch master models with different implants (Straumann Bone Level (BL), and Standard Plus Tissue Level (TL) Straumann, Basel, Switzerland). The authors concluded that for the BL test groups, CI presented significantly lower distortion than DI. In a similar study, Chia *et al.* (30) compared the accuracy of CI versus DI. The authors concluded that CI with 0º angulation between implants was associated with the highest accuracy, although no significant differences were found between different angulations when comparing CI and DI

In Vitro - CE.

In 2016, Papaspyridakos *et al.* (32) did not find significant differences between CI and DI compared with the master cast, with exception of Group II [(Open-Tray non-splinted at implant level) (OPNSPT-BL)]. Menini *et al.* (34) used a CE model with four low-profile implant analogs to evaluate impression accuracy in four different groups: CI (open tray-splinted vs. open tray-no splinted vs. closed tray) and DI (PEEK scanbody, True Definition [3M ESPE]). The authors found that DI achieved higher accuracy than CI. Amin *et al.* (33) used a mandibular model with five inter-foramen analogs in a stone master cast to compare the accuracy of CI and DI, concluding that DI was significantly more accurate than CI.

## Discussion

This systematic review was designed to evaluate the accuracy and efficiency of IOS for dental implant impression taking, compared with CI, and to assess the economic feasibility of introducing digital techniques.

The *in vivo* evidence located in the first search was scarce, further reduced by risk of bias determined by the CASP quality assessment (8 studies). So in order to expand the amount of information on the topic, an additional search was carried out expanding the criteria to include *in vitro* studies. In order to critically appraise the works identified, the authors adapted a previously published checklist18 for assessing the potential bias of *in vitro* studies. This checklist was initially designed to evaluate the quality of *in vitro* studies investigating dental materials. However, applying the checklist to the studies selected in the present review, none fulfilled points 5 to 9. Point 5 of this checklist analyzes sample size, while points 6-9 analyze randomization (sequence generation, allocation concealment mechanism, implementation, and blinding). An *in vitro* study which evaluates dental implant impression-taking employs a previously designed model, with replicas of dental implants from which impressions are taken. The choice of model does not alter the results, as the models are manufactured industrially in advance and so the rate of error from model to model is negligible. In turn, there is no need for randomization, and sample size does not affect the results obtained. In this way, the authors of the present review used a modified version of the checklist published in 2012 by Faggion *et al*. (35), removing questions 5-9. In this way, the risk of bias and the quality of the *in vitro* studies analyzed were assessed by an appropriate, simple, and practical method.

Because of the variability between the *in vivo* studies included and the fact that it was unclear how passive fit had been evaluated, comparisons of the results were not possible (11,23-26). Likewise, the *in vitro* studies reviewed could not be compared because of the different methods and IOS employed in both partial (27-31) and completely edentulous model (32-34). Nevertheless, most of the studies analyzed obtained results indicating sufficient accuracy, precision or trueness to guarantee adequate passive fit; especially on partially edentulous models. Several authors concluded that dental implant angulation and depth did not influence outcomes in terms of passive fit (15-17). Regarding the economic feasibility of DI, in comparisons between DI and CI, only a single *in vivo* study found that DI allowed a more efficient workflow than CI (23).

Nevertheless, four systematic reviews have been conducted evaluating if there are any significant differences in accuracy between CI and DI (one *in vitro* study (36), two *in vivo* (37,38) studies and one that analyzed both *in vivo* and *in vitro* (39)studies) and all authors have concluded that the quality and quantity of the articles analyzed were insufficient. The present systematic review studied the same issue, analyzing both *in vivo* and *in vitro* studies, and adding one further objective, to determine the economic feasibility of DI.

## Conclusions

Based on the data extracted from the articles analyzed in this systematic review, objectives could not be clearly and objectively addressed. It was not possible to determine which implant impression technique leads to better passive fit of superstructures. Digital techniques with intraoral scan impressions offer promising results, although improvements are still needed, particularly in full-arch impression taking. The available *in vivo* evidence is scarce, mainly case reports, which only provided low quality evidence. Randomized clinical studies comparing conventional and digital implant impression techniques are needed to generate decisive evidence. Finally, insufficient evidence was found regarding the economic feasibility of DI for implant-supported restorations, so additional research is needed to clarify this.
